# Associated factors for multidimensional attitudes and behaviors of reproductive health toward pregnancy among early and late adolescents in Tanzania: a cross-sectional study

**DOI:** 10.1186/s12978-023-01583-2

**Published:** 2023-03-14

**Authors:** Naoki Hirose, Chen Sanmei, Mariko Okamoto, Frida E. Madeni, Nicolaus Madeni, Ayaka Teshima, Yasunobu Ando, Koji Takahama, Mayu Yoshikawa, Yu Kunimoto, Yoko Shimpuku

**Affiliations:** 1 Graduate School of Biomedical and Health Sciences, Hiroshima University, 1-2-3, Kasumi, Minamiku, Hiroshima 734-8553 Japan; 2 Tokyo, Japan; 3 Korogwe Town Council, Tanga, Tanzania; 4 Imperial College London, St Mary’s Campus, Norfolk Place, London, W2 1PG UK; 5 National Institute of Advanced Industrial Science and Technology, Tokyo, Japan; 6 Beyond Next Ventures, Tokyo, Japan

**Keywords:** Reproductive health, Adolescent pregnancy, Tanzania, Risk factor analyses

## Abstract

**Background:**

Adolescent pregnancy is a serious reproductive health problem in Tanzania. However, the risk factors for multidimensional attitudes and behaviors of reproductive health toward pregnancy in Tanzanian adolescents remain unexplored.

**Methods:**

We collected baseline characteristics and information on attitudes and behaviors of reproductive health from 4161 Tanzanian adolescents in all 54 primary and secondary schools in the Korogwe district. We applied mixed effect multiple regression analyses stratified by sex to find the factors related to reproductive health attitudes and behaviors toward pregnancy.

**Results:**

In female students, regarding the attitudes of reproductive health, higher age, hope for marriage in the future, a talk with a parent about sex or pregnancy, and a higher hope score were significantly associated with a lower score. For the behaviors of reproductive health, higher age, a talk with a parent about sex or pregnancy, time to talk with a parent about daily life, and a higher hope score were significantly associated with a lower score. In male students, regarding the attitudes of reproductive health, a higher hope score was significantly associated with a lower score. For the behaviors of reproductive health, higher age, time to talk with a parent about daily life, and a higher hope score was significantly associated with a lower score.

**Conclusions:**

The heterogeneous factor-outcomes association between female and male students suggested that sex-specialized interventions may be required to change their risky attitudes or behaviors of reproductive health. Although we cannot conclude as points of intervention, our study suggested that it may be practical to improve parent-adolescents communication about sex or reproductive health and change adolescents’ views of pregnancy or marriage for gaining financial or social status.

## Introduction

Approximately 12.1 million girls aged 15–19 years give birth each year in developing countries [1]. Especially in Africa, with the inaccessibility of contraceptive methods, the unfavorable attitudes of the community toward adolescent contraceptive use, and poor knowledge of sexual and reproductive health in adolescents, the prevalence of adolescent pregnancy is higher at 18.8% compared to other low- to middle-income countries (6.4% in Latin America, 4.5% Southeastern Asia, and 0.7% in Eastern Asia) [2]. Moreover, half of the adolescent pregnancies happened unintendedly in girls aged 15–19 in developing countries [1]. Adolescent pregnancy brings unfavorable consequences to girls from physical, mental, and social perspectives [3]. World Health Organization reported that pregnancy and childbirth complications are the leading cause of death for girls aged 15–19 years. In addition, 5.6 million abortions are estimated to occur annually, of which 3.9 million are unsafe, leading to maternal mortality, morbidity, and lasting health problems [3]. It is also reported that adolescent pregnancy is a high risk factor for depressive symptoms, increasing the risk for substance and alcohol abuse and a harsher parenting style in adolescents [4].

Moreover, adolescent pregnancy increases school dropout rates, leading to economic and social disadvantages [5]. Approximately 50% of teen mothers receive a high school diploma [6]. Due to these physical, mental, and social disadvantages that girls suffer from adolescent pregnancy, the decrease in adolescent pregnancy rate is set as one of the global Sustainable Development Goals (3.7.2) [7].

In Tanzania, the adolescent pregnancy rate is very high at 27% [8], with the ninth highest frequency worldwide [9]. Between 2010 and 2015–16, a 4% increase in the pregnancy rate was observed in Tanzanian teenage [10]. Although policy change in late 2021 in Tanzania allowed teen mothers who dropped out from school to return, school dropout due to pregnancy is still a significant issue to solve. Therefore, finding the risk factors that improve or worsen reproductive health attitudes and behaviors for effective pregnancy prevention among adolescents in Tanzania is critically required.

However, the risk factors of reproductive health attitudes and behaviors toward pregnancy among adolescents in Tanzania remain unexplored. First, previous studies about adolescent sexual and reproductive health mainly focused on older adolescents. Only a few studies have been conducted for early adolescents from 10 to 14 years in Sub-Saharan Africa, including Tanzania [11]. Second, most of these studies focused on narrowly defined outcomes of reproductive health attitudes and behaviors, such as condom use or the sexual intercourse experience [12]. However, these outcomes reflected only one side of adolescent reproductive health attitudes and behaviors. Risk factor analyses with the outcomes of reproductive health attitudes and behaviors measured through multidimensional aspects enable a more comprehensive understanding of the association between adolescents' characteristics and reproductive health behaviors. Third, among all studies regarding reproductive health attitudes and behaviors among adolescents in Tanzania, only one explored the risk factors involving reproductive health attitudes and behaviors [12]. That study detected being male, young age, and being in school as risk factors for dangerous sexual behaviors. However, many potential factors remain to be investigated that may affect reproductive health attitudes and behaviors in adolescents, such as communication on sexual topics with a parent, experience of sexual intercourse, or future career plans [12]. In addition, this previous study did not divide the analytic cohort into females and males. However, it has been reported that female and male adolescents have different reproductive behaviors [13]. Therefore, female and male adolescents may have distinct mechanisms relating to risk factors and reproductive health behaviors. To address this effect modification by sex, subgroup analyses by sex should be conducted.

The “adolescent education project to prevent social isolation due to unwanted pregnancy and school dropouts” was implemented in Tanzania by the Japanese NPO Class for everyone, including the local NGO, the New Rural Children Foundation, as a JICA Partnership Program. In this study, a baseline survey was conducted to contextualize reproductive health attitudes in adolescents. This survey collected several baseline characteristics, attitudes, and behaviors of reproductive health among early and late adolescents in Tanzania, which was unaddressed in previous studies. This study aimed to explore risk factors of multidimensional attitudes and reproductive health behaviors among Tanzanian adolescents.

## Methods

We performed a cross-sectional study using primarily collected data from children in primary and secondary schools in Tanzania.

### The study setting

Data were collected in all 41 primary and 13 secondary schools in three wards of Korogwe District, an area in Northeast Tanzania [14]. These three wards were selected from rural, urban, and middle, respectively to make a representative population in Korogwe District. Korogwe is located between two major cities, Dar es Salaam and Arusha. The population is 242,038 and the total area is 3293 km^2^ in Korogwe, where there is one hospital, 3 health centers, and 48 dispensaries.

### Study population and sampling

The target of the baseline survey was the whole 5th to 6th-grade students in primary schools and 1st and 2nd-grade students in secondary schools. Students in the final grade of both primary and secondary schools (7th grade in primary schools and 3rd and 4th grades in secondary school) were not intendedly to be included to minimize the missing after graduation because this study was conducted as a part of the longitudinal intervention study. The project team had a meeting with all school principals in the three wards and explained the project to request their cooperation. Subsequently, upon permission from the local council, the project team visited schools and explained the projects' details to teachers. Finally, with the cooperation of teachers, the project team explained the project and data collection to the whole target students. We included all students who: (1) were in the grades described above and (2) agreed to participate in the study. We excluded three students whose sex was missing because sex was an essential variable in making subgroup cohorts by sex.

### Data collection

We collected data from 2852 students who met the eligibility criteria from primary schools and 1309 students from secondary schools. The questionnaires were distributed to all eligible students by investigators or teachers in each school, and students themselves answered the questionnaire. To minimize missing answers, for each missing or inappropriate item, investigators or teachers contacted students. They guided them to fill in the answers, excluding the case when students intentionally avoided answering these items. Investigators explained to teachers the purpose and contents of the survey, and then teachers explained them to students. Students provided informed consent to attend the survey. This study received ethical approval from the National Institute of Medical Research in Tanzania (NIMR/HQ/R.8a/Vol.IX988).

### Outcomes

The outcome of this study was multidimensional attitudes and behaviors of reproductive health, which were measured by the instrument originally developed by Madeni et al. [15]. The authors reported that two experienced nurse researchers assessed content validity in the instrument development process. Also, the instrument was pilot tested on a group of 30 students in Dar es Salaam, and they did not experience any issues with the instrument. The attitudes measurement consisted of eight items, and students selected one from five options: (1) strongly disagree; (2) somewhat disagree; (3) either agree or disagree; (4) somewhat agree; (5) strongly agree. The total score was calculated by adding the score of all items, which ranged from 8 to 40 points. A higher score indicated that students could escape from situations that expose them to the danger of unwanted pregnancy or infection with HIV/AIDS. The behaviors measurement consisted of six items, and the options for each item were similar to those of the attitude measurement. The total score ranged from 6 to 30. A higher score indicated that students had made good decisions to say no to sexual behaviors. The list of items for these measurements is described in Table 1.

**Table 1:** Items of attitudes or behaviors of reproductive health measurement and hope measurement

Attitudes of reproductive health measurement
It is okay for girls to say no when they don't want to be touched by boys
It is good to have babies during adolescence (reverse item)
It is dangerous to accept someone's lift when going and coming back from school
I better concentrate only on my studies; love will come later
It is not good that some adults, especially men, take advantage of their wealth or position in society to pressure young people, especially girls, to have sex with them
I better engage in sexual activities with an older man to get money when we don't have enough money at home (reverse item)
I believe reproductive health education for young people will increase their sexual activities (reverse item)
I believe girls accept sex only because they want gifts or money (reverse item)

Behaviors of reproductive health measurement
I can avoid becoming pregnant if I can avoid sex or use intact condoms
I will not have sex with a boy who is my friend
I put boys under pressure to have sex
I want to have sex with my boyfriend before marriage because I love him (reverse item)
If I don’t want to have sexual intercourse, but my friends or partner forces me to do so, I will agree (reverse item)
I will say yes to sex because it is a part of our life (reverse item)

Hope measurement
I have a positive outlook toward life
I have short and/or long-range goals
I feel all alone (reverse item)
I can see possibilities in the midst of difficulties
I have a faith that gives me comfort
I feel scared about my future (reverse item)
I can recall happy/joyful times
I have deep inner strength
I am able to give and receive caring/love
I have a sense of direction
I believe that each day has potential
I feel my life has value and worth

### Covariates

We collected information on the following baseline characteristics of adolescents via the questionnaire: age (< 12, 12–14, > 14), having brothers or sisters (yes or no), religion (Christian, Muslim, or others), the experience of menstruation or nocturnal emission (yes or no), the experience of sexual intercourse (yes or no), hope for marriage in the future (yes or no), ever talked with a parent about sex or pregnancy (yes or no), have time to talk with a parent about daily life (yes or no), and hope for the future. Hope in young people was measured by the Herth Hope Index (HHI), designed by Herth [16]. The HHI consisted of 12 items, and each of them had been rated on a 4-point Likert scale: (1) strongly agree; (2) agree; (3) disagree; (4) strongly disagree. The total score was calculated by adding the score of each item. A higher score indicated that students had a stronger hope. The list of items for the hope score is described in Table 1. HHI showed high divergent validity with other established scale and robust construct validity. Moreover, we assigned the school's place (town or rural) and stage (primary or secondary) to each student from the school data where the survey was conducted.

### Statistical analyses

Participants’ outcomes and characteristics were described with mean and standard deviation for continuous variables and percentage and interquartile range for categorical variables.

We conducted multiple regression analyses to find the risk factors of the attitudes and behaviors of reproductive health. All analyses were stratified by sex because the association between covariates and outcomes would vary depending on sex. We included all covariates in these analyses. We also included squared terms for age because the ad-hoc analysis showed a non-linear association between age and the attitudes and behaviors of reproductive health. In the multiple regression analyses, we included a random effect of school to consider the intra-school correlation. Participants who had missing data were excluded from the analyses. We confirmed collinearity in these multiple regression analyses by checking the variance inflationary factor (VIF) for included covariates.

To check the robustness of the analyses, we conducted a sensitivity analysis, where we applied the multiple imputation method considering the risk of selection bias caused by excluding the participants who had missing data. We used the chained equations to create ten imputed datasets (mice; multivariate imputation by chained equations in R) for the multiple imputations. The estimates from the 10 imputed datasets were then combined using Rubin’s rules [17]. We assumed missing at random for missing observations for covariates. Also, we conducted a subgroup analysis by school level (primary school or secondary school), to consider the potential effect modification by school level. In these analyses, we did not include age as a covariate because it was assumed that age was highly correlated with school level.

All tests were two-tailed, and the threshold of significance was a P-value of < 0.05. We used R version 4.1.3 for all analyses.

## Results

Table 2 shows the characteristics of adolescents. Out of the total adolescents (N = 4161), 68.5% were from primary schools, and 31.5% were from secondary schools, of which 67.8% schools were in town and 32.2% were in rural. Students aged 12–14 years were 43.0% and below 14 years were 41.6%. Among the participants, 53.7% were female, 76.4% answered that they had hope for marriage in the future, and 30.4% had an experience of menstruation or nocturnal emission. Only 0.6% had an experience sexual intercourse. Students who had ever talked with a parent about sex or pregnancy were 40.4%, and 83.4% had time to talk with a parent about daily life. The hope score was 39.0 (standard deviation (SD): 4.8). The attitudes and behaviors of reproductive health scores were 18.6 (SD: 5.2) and 14.7 (SD: 3.3), respectively.

**Table 2:** Baseline characteristics and outcomes

Level	Overall	Female	Male	P-value
	n = 4161	n = 2234	n = 1924	
School (%)				
Secondary	1309 (31.5)	719 (32.2)	590 (30.7)	0.309
Primary	2852 (68.5)	1515 (67.8)	1334 (69.3)	
Place (%)				
Rural	1338 (32.2)	716 (32.1)	622 (32.3)	0.874
Town	2823 (67.8)	1518 (67.9)	1302 (67.7)	
Age (%)				
− 12	617 (14.8)	419 (18.8)	197 (10.2)	< 0.001
12–14	1789 (43.0)	985 (44.1)	803 (41.7)	
14-	1731 (41.6)	825 (36.9)	905 (47.0)	
Missing	24 (0.6)	5 (0.2)	19 (1.0)	
Have brother/sister (%)				
No	487 (11.7)	235 (10.5)	252 (13.1)	0.025
Yes	3652 (87.8)	1989 (89.0)	1660 (86.3)	
Missing	22 (0.5)	10 (0.4)	12 (0.6)	
Religion (%)				
Christian	1668 (40.1)	911 (40.8)	754 (39.2)	0.521
Muslim	2440 (58.6)	1293 (57.9)	1147 (59.6)	
Others	27 (0.6)	17 (0.8)	10 (0.5)	
Missing	26 (0.6)	13 (0.6)	13 (0.7)	
Hope for marriage (%)				
No	949 (22.8)	599 (26.8)	349 (18.1)	< 0.001
Yes	3177 (76.4)	1624 (72.7)	1551 (80.6)	
Missing	35 (0.8)	11 (0.5)	24 (1.2)	
Experience of menstruation or nocturnal emission (%)				
No	2863 (68.8)	1555 (69.6)	1306 (67.9)	0.353
Yes	1265 (30.4)	664 (29.7)	600 (31.2)	
Missing	33 (0.8)	15 (0.7)	18 (0.9)	
Experience of sexual intercourse (%)				
No	4104 (98.6)	2210 (98.9)	1891 (98.3)	0.006
Yes	26 (0.6)	6 (0.3)	20 (1.0)	
Missing	31 (0.7)	18 (0.8)	13 (0.7)	
Talk about sex or pregnancy with parents (%)				
No	2459 (59.1)	1283 (57.4)	1175 (61.1)	0.003
Yes	1679 (40.4)	944 (42.3)	733 (38.1)	
Missing	23 (0.6)	7 (0.3)	16 (0.8)	
Have daily talk with parents (%)				
No	672 (16.1)	373 (16.7)	299 (15.5)	0.007
Yes	3469 (83.4)	1857 (83.1)	1609 (83.6)	
Missing	20 (0.5)	4 (0.2)	16 (0.8)	
The hope for the future (mean (SD))	39.0 (4.8)	39.0 (5.0)	39.0 (4.5)	0.56
The attitudes of reproductive health score (mean (SD))	18.6 (5.2)	18.4 (5.2)	18.9 (5.2)	0.002
The behaviors of reproductive health score (mean (SD))	14.7 (3.3)	14.3 (3.3)	15.2 (3.3)	< 0.001

Table 3 shows the results of mixed-effect multiple regression analyses stratified by sex. In female students, for the attitudes of reproductive health, higher age, hope for marriage in the future, having a talk with a parent about sex or pregnancy, a higher hope score was significantly associated with a lower score. For the behaviors of reproductive health, higher age, having a talk with a parent about sex or pregnancy, having time to talk with a parent about daily life, and a higher hope score was significantly associated with a lower score. In male students, for the attitudes of reproductive health, a higher hope score was significantly associated with a lower score. For the attitudes of reproductive health, higher age, having time to talk with a parent about daily life, and a higher hope score was significantly associated with a lower score. In all models, there was no evidence of collinearity between variables with all VIF values < 2. In sensitivity analyses where we conducted multiple imputations for missing data (Table 4), we confirmed the same tendency, excluding the experience of menstruation or nocturnal emission that was significantly associated with a higher score in the behaviors of reproductive health in male students. Table 5 shows the results of the subgroup analysis by school level. The results in the subgroup of primary school were similar to those in the subgroup of secondary school, which indicated that the school level may not alter our conclusions.

**Table 3:** Results of mixed-effect multiple regression analyses

Attitudes of reproductive health									
Female					Male				
	Coefficient	95% CI		P-value		Coefficient	95% CI		P-value
Place					Place				
Rural	Reference				Rural	Reference			
Town	− 0.15	− 0.99	0.69	0.72	Town	− 0.72	− 1.81	0.36	0.20
Age					Age				
− 12	Reference				− 12	Reference			
12–14	− 0.39	− 1.00	0.23	0.22	12–14	− 0.07	− 0.90	0.76	0.87
14−	− 0.94	− 1.75	− 0.13	0.02	14−	− 0.30	− 1.21	0.61	0.52
Have brother/sister					Have brother/sister				
No	Reference				No	Reference			
Yes	− 0.10	− 0.80	0.60	0.78	Yes	0.60	− 0.09	1.28	0.09
Religion					Religion				
Christian	Reference				Christian	Reference			
Muslim	0.19	− 0.26	0.64	0.41	Muslim	− 0.23	− 0.73	0.26	0.36
Others	0.23	− 2.54	3.00	0.87	Others	− 0.24	− 3.52	3.04	0.89
Hope for marriage					Hope for marriage				
No	Reference				No	Reference			
Yes	− 0.85	− 1.34	− 0.35	< 0.001	Yes	− 0.45	− 1.06	0.17	0.15
Experience of menstruation or nocturnal emission					Experience of menstruation or nocturnal emission				
No	Reference				No	Reference			
Yes	− 0.09	− 0.66	0.48	0.76	Yes	0.33	− 0.23	0.88	0.25
Experience of sexual intercourse					Experience of sexual intercourse				
No	Reference				No	Reference			
Yes	0.36	− 3.52	4.25	0.85	Yes	0.63	− 1.86	3.12	0.62
Hope in the future	− 0.18	− 0.23	− 0.14	< 0.001	Hope in the future	− 0.17	− 0.22	− 0.11	< 0.001
Talk about sex or pregnancy with parents					Talk about sex or pregnancy with parents				
No	Reference				No	Reference			
Yes	− 0.52	− 0.98	− 0.07	0.02	Yes	0.21	− 0.29	0.70	0.41
Have daily talk with parents					Have daily talk with parents				
No	Reference				No	Reference			
Yes	− 0.52	− 1.10	0.06	0.08	Yes	− 0.05	− 0.70	0.59	0.87

Behaviors of reproductive health									
Female					Male				
	Coefficient	95% CI		P-value		Coefficient	95% CI		P-value
Place					Place				
Rural	Reference				Rural	Reference			
Town	− 0.05	− 0.70	0.59	0.87	Town	0.14	− 0.61	0.89	0.72
Age					Age				
− 12	Reference				− 12	Reference			
12–14	− 0.03	− 0.41	0.36	0.90	12–14	− 0.40	− 0.92	0.12	0.14
14−	− 0.62	− 1.15	− 0.10	0.02	14−	− 0.64	− 1.21	− 0.06	0.03
Have brother/sister					Have brother/sister				
No	Reference				No	Reference			
Yes	− 0.07	− 0.51	0.36	0.74	Yes	− 0.21	− 0.63	0.22	0.34
Religion					Religion				
Christian	Reference				Christian	Reference			
Muslim	0.004	− 0.28	0.29	0.98	Muslim	0.15	− 0.16	0.46	0.34
Others	− 0.50	− 2.23	1.22	0.57	Others	0.85	− 1.20	2.91	0.42
Hope for marriage					Hope for marriage				
No	Reference				No	Reference			
Yes	− 0.30	− 0.61	0.01	0.06	Yes	− 0.05	− 0.43	0.34	0.81
Experience of menstruation or nocturnal emission					Experience of menstruation or nocturnal emission				
No	Reference				No	Reference			
Yes	0.09	− 0.27	0.45	0.61	Yes	0.34	− 0.01	0.69	0.05
Experience of sexual intercourse					Experience of sexual intercourse				
No	Reference				No	Reference			
Yes	0.02	− 2.41	2.44	0.99	Yes	0.81	− 0.75	2.37	0.31
Hope in the future	− 0.07	− 0.10	− 0.04	< 0.001	Hope in the future	− 0.05	− 0.08	− 0.01	0.01
Talk about sex or pregnancy with parents					Talk about sex or pregnancy with parents				
No	Reference				No	Reference			
Yes	− 0.49	− 0.77	− 0.20	0.001	Yes	− 0.17	− 0.48	0.14	0.28
Have daily talk with parents					Have daily talk with parents				
No	Reference				No	Reference			
Yes	− 0.53	− 0.89	− 0.16	0.004	Yes	− 0.61	− 1.01	− 0.21	0.003

**Table 4:** Results of mixed-effect multiple regression analyses with multiple imputations

Attitudes of reproductive health									
Female					Male				
	Coefficient	95% CI		P-value		Coefficient	95% CI	P-value	
Place					Place				
Rural	Reference				Rural	Reference			
Town	− 0.15	− 0.94	0.65	0.72	Town	− 0.65	− 1.70	0.39	0.22
Age					Age				
− 12	Reference				− 12	Reference			
12–14	− 0.57	− 1.17	0.03	0.06	12–14	− 0.01	− 0.82	0.80	0.98
14−	− 1.20	− 2.00	− 0.41	0.003	14−	− 0.24	− 1.12	0.63	0.58
Have brother/sister					Have brother/sister				
No	Reference				No	Reference			
Yes	− 0.03	− 0.71	0.65	0.93	Yes	0.57	− 0.09	1.23	0.09
Religion					Religion				
Christian	Reference				Christian	Reference			
Muslim	0.11	− 0.34	0.56	0.63	Muslim	− 0.25	− 0.73	0.23	0.31
Others	0.59	− 1.84	3.02	0.63	Others	− 0.35	− 3.45	2.75	0.82
Hope for marriage					Hope for marriage				
No	Reference				No	Reference			
Yes	− 0.76	− 1.24	− 0.28	0.002	Yes	− 0.36	− 0.95	0.24	0.24
Experience of menstruation or nocturnal emission					Experience of menstruation or nocturnal emission				
No	Reference				No	Reference			
Yes	− 0.10	− 0.67	0.46	0.72	Yes	0.27	− 0.25	0.80	0.31
Experience of sexual intercourse					Experience of sexual intercourse				
No	Reference				No	Reference			
Yes	0.06	− 3.85	3.97	0.98	Yes	0.51	− 1.70	2.72	0.65
Hope in the future	− 0.19	− 0.24	− 0.14	< 0.001	Hope in the future	− 0.17	− 0.23	− 0.12	< 0.001
Talk about sex or pregnancy with parents					Talk about sex or pregnancy with parents				
No	Reference				No	Reference			
Yes	− 0.47	− 0.92	− 0.03	0.04	Yes	0.20	− 0.27	0.67	0.41
Have daily talk with parents					Have daily talk with parents				
No	Reference				No	Reference			
Yes	− 0.53	− 1.10	0.03	0.06	Yes	− 0.21	− 0.84	0.42	0.52

Behaviors of reproductive health									
Female					Male				
	Coefficient	95% CI		P-value		Coefficient	95% CI		P-value
Place					Place				
Rural	Reference				Rural	Reference			
Town	− 0.07	− 0.69	0.56	0.84	Town	0.03	− 0.68	0.74	0.93
Age					Age				
− 12	Reference				− 12	Reference			
12–14	− 0.04	− 0.40	0.32	0.84	12–14	− 0.37	− 0.86	0.12	0.14
14−	− 0.70	− 1.20	− 0.19	0.01	14−	− 0.63	− 1.17	− 0.08	0.02
Have brother/sister					Have brother/sister				
No	Reference				No	Reference			
Yes	− 0.16	− 0.57	0.26	0.46	Yes	− 0.18	− 0.60	0.24	0.41
Religion					Religion				
Christian	Reference				Christian	Reference			
Muslim	0.01	− 0.26	0.29	0.92	Muslim	0.15	− 0.15	0.45	0.32
Others	− 0.44	− 1.95	1.08	0.57	Others	0.68	− 1.27	2.63	0.49
Hope for marriage					Hope for marriage				
No	Reference				No	Reference			
Yes	− 0.24	− 0.55	0.07	0.13	Yes	− 0.09	− 0.47	0.28	0.63
Experience of menstruation or nocturnal emission					Experience of menstruation or nocturnal emission				
No	Reference				No	Reference			
Yes	0.10	− 0.25	0.45	0.59	Yes	0.38	0.03	0.74	0.03
Experience of sexual intercourse					Experience of sexual intercourse				
No	Reference				No	Reference			
Yes	− 0.13	− 2.55	2.29	0.92	Yes	0.55	− 0.85	1.95	0.44
Hope in the future	− 0.08	− 0.10	− 0.05	< 0.001	Hope in the future	− 0.05	− 0.08	− 0.01	0.01
Talk about sex or pregnancy with parents					Talk about sex or pregnancy with parents				
No	Reference				No	Reference			
Yes	− 0.44	− 0.72	− 0.16	0.002	Yes	− 0.15	− 0.45	0.15	0.32
Have daily talk with parents					Have daily talk with parents				
No	Reference				No	Reference			
Yes	− 0.47	− 0.83	− 0.11	0.01	Yes	− 0.59	− 0.99	− 0.18	0.005

**Table 5:** Results of mixed-effect multiple regression analyses with multiple imputation by school level

Attitudes of reproductive health									
Primary school					Secondary school				
	Coefficient	95% CI		P-value		Coefficient	95% CI		P-value
Place					Place				
Rural	Reference				Rural	Reference			
Town	− 0.52	− 1.31	0.26	0.19	Town	0.24	− 0.99	1.46	0.70
Have brother/sister					Have brother/sister				
No	Reference				No	Reference			
Yes	− 0.04	− 0.65	0.57	0.89	Yes	0.44	− 0.26	1.15	0.22
Religion					Religion				
Christian	Reference				Christian	Reference			
Muslim	− 0.05	− 0.46	0.36	0.8	Muslim	− 0.02	− 0.54	0.51	0.95
Others	0.05	− 2.3	2.4	0.97	Others	− 0.55	− 3.81	2.72	0.74
Hope for marriage					Hope for marriage				
No	Reference				No	Reference			
Yes	− 0.19	− 0.62	0.24	0.38	Yes	− 1.61	− 2.39	− 0.84	< 0.001
Experience of menstruation or nocturnal emission					Experience of menstruation or nocturnal emission				
No	Reference				No	Reference			
Yes	0.61	0.09	1.14	0.02	Yes	− 0.07	− 0.62	0.48	0.80
Experience of sexual intercourse					Experience of sexual intercourse				
No	Reference				No	Reference			
Yes	0.75	− 1.38	2.89	0.49	Yes	− 1.76	− 5.87	2.35	0.40
Hope in the future	− 0.18	− 0.22	− 0.13	< 0.001	Hope in the future	− 0.16	− 0.22	− 0.1	< 0.001
Talk about sex or pregnancy with parents					Talk about sex or pregnancy with parents				
No	Reference				No	Reference			
Yes	− 0.33	− 0.74	0.08	0.12	Yes	0.18	− 0.36	0.73	0.51
Have daily talk with parents					Have daily talk with parents				
No	Reference				No	Reference			
Yes	− 0.24	− 0.74	0.26	0.35	Yes	− 0.73	− 1.5	0.05	0.07

Behaviors of reproductive health									
Primary school					Secondary school				
	Coefficient	95% CI		P-value		Coefficient	95% CI		P-value
Place					Place				
Rural	Reference				Rural	Reference			
Town	− 0.01	− 0.46	0.44	0.97	Town	0.34	− 0.83	1.50	0.57
Have brother/sister					Have brother/sister				
No	Reference				No	Reference			
Yes	− 0.22	− 0.64	0.20	0.30	Yes	− 0.23	− 0.62	0.15	0.23
Religion					Religion				
Christian	Reference				Christian	Reference			
Muslim	0.00	− 0.27	0.27	0.99	Muslim	0.18	− 0.10	0.47	0.21
Others	− 0.54	− 2.07	0.99	0.49	Others	0.41	− 1.35	2.17	0.65
Hope for marriage					Hope for marriage				
No	Reference				No	Reference			
Yes	− 0.07	− 0.35	0.21	0.62	Yes	0.11	− 0.31	0.53	0.61
Experience of menstruation or nocturnal emission					Experience of menstruation or nocturnal emission				
No	Reference				No	Reference			
Yes	0.70	0.34	1.06	< 0.001	Yes	0.00	− 0.29	0.3	0.98
Experience of sexual intercourse					Experience of sexual intercourse				
No	Reference				No	Reference			
Yes	0.41	− 1.02	1.85	0.57	Yes	1.51	− 0.71	3.73	0.18
Hope in the future	− 0.07	− 0.1	− 0.04	< 0.001	Hope in the future	− 0.03	− 0.07	0.00	0.04
Talk about sex or pregnancy with parents					Talk about sex or pregnancy with parents				
No	Reference				No	Reference			
Yes	− 0.18	− 0.45	0.08	0.18	Yes	− 0.62	− 0.91	− 0.32	< 0.001
Have daily talk with parents					Have daily talk with parents				
No	Reference				No	Reference			
Yes	− 0.45	− 0.78	− 0.12	0.01	Yes	− 0.41	− 0.83	0.01	0.06

## Discussion

We conducted multiple regression analyses to find the risk factors of multidimensional attitudes and behaviors of reproductive health among Tanzanian adolescents. We found several risk factors significantly related to risky attitudes or behaviors of reproductive health, which could lead to adolescent pregnancy. This study adds to the existing evidence by measuring attitudes and behaviors of reproductive health with multidimensional scales from a large sample size, including early adolescents, stratifying the cohort by sex to consider the effect modification.

To the best of our knowledge, we were aware of only one study that explored risk factors of the attitudes or behaviors of reproductive health in Tanzanian adolescents [18]. However, that particular study included limited potential risk factors such as sex, age, school status, and religion. In addition, that study conducted the analyses with the whole population without separating female and male students, even though female and male students could have different mechanisms relating to risk factors and attitudes or behaviors of reproductive health. Contrarily, our study included a broader perspective than the previous study and stratified the cohort by sex to address the effect modification due to sex in the association between adolescents’ characteristics and attitudes or behaviors of reproductive health.

As hypothesized, we found a heterogeneous association of risk factors with the attitudes or behaviors of reproductive health between male and female students. In female students, having a talk with a parent about sex or pregnancy was significantly associated with risky attitudes or behaviors regarding reproductive health, unlike in male students. Previous studies conducted in low- and middle-income countries (LMIC) reported a similar inconsistent impact of parent-adolescent communication on sexual activities between female and male adolescents [19, 20]. A systematic review paper about parent-adolescent communication in LMIC reported that the impact of parent-adolescent communication on sexual intentions, behaviors, and contraceptive use is inconclusive [21]. Some studies found harmful effects of parent-adolescent communication, such as a higher sexual intercourse rate in the last 12 months [22–24]. Other studies found protective effects of parent-adolescent communication, such as higher contraceptive use [22, 25] and delayed sexual debut [26]. This inconsistency in previous studies may be attributed to contents, quality, and frequency of parent-adolescent communication [21, 27]. A previous study in Tanzanian villages suggested that the concept of “care” by parents for children had wider diversity across villages [28]. While this study did not collect information on the value of parents regarding sex or pregnancy and the concept of “care” in study settings, parents’ values regarding pregnancy could potentially affect the attitudes or behaviors of reproductive health in children. Therefore, our study result, which indicated parent-adolescent communication impact about reproductive health in female students toward pregnancy and no strong evidence for the favorable impact on male students, may suggest the necessity to improve parent-adolescent communication or knowledge of parents concerning reproductive health.

In female and male students, a higher hope score was significantly associated with the attitudes or behaviors of reproductive health toward pregnancy. The tradition of child marriage in Tanzania may explain this finding. A qualitative study conducted in rural Tanzania reported that marriage is viewed as a way of becoming an adult, gaining status within the community, and providing financial support and pride to their families [29]. Therefore, in our study, the proactive attitudes or behaviors toward marriage or pregnancy may have been reflected in a better expectation for the future. It may suggest changing their view of marriage to obtain social and financial status to prevent adolescent pregnancy.

Several limitations should be acknowledged while interpreting our study results. First, this was a cross-sectional study that aimed to find potential risk factors for the attitudes and behaviors of reproductive health toward pregnancy in Tanzanian adolescents. We need further studies with designs and methods suited for causal inference to conclude causation between these factors. Second, this study was conducted in one district of Tanzania. The generalizability of study results to adolescents in other districts in Tanzania or other LMICs may be limited if there are effect modifiers.

## Conclusion

We found several risk factors associated with the multidimensional attitudes or behaviors of reproductive health toward pregnancy among Tanzanian adolescent girls and boys. The heterogeneity between female and male students in the associated factors with attitudes and behaviors of reproductive health suggested that sex-specific interventions may be required to improve attitudes or behaviors of reproductive health. In addition, although we cannot conclude, as points of intervention, our study suggested that it is of great practical significance to improve parent-adolescent communications on sexual and reproductive health and change adolescents’ views on early marriage and teenager pregnancy for obtaining financial or social status.

## Data Availability

The dataset used and analyzed during the current study is available from the corresponding author upon reasonable request.

## References

[1] Darroch JE, Singh S (2013). Trends in contraceptive need and use in developing countries in 2003, 2008, and 2012: an analysis of national surveys. Lancet.

[2] Kassa GM, Arowojolu AO, Odukogbe AA, Yalew AW (2018). Prevalence and determinants of adolescent pregnancy in Africa: a systematic review and meta-analysis 11 medical and health sciences 1117 public health and health services. Reprod Health.

[3] World Health Organization. Adolescent pregnancy. https://www.who.int/en/news-room/fact-sheets/detail/adolescent-pregnancy. Geneva: WHO;2020.

[4] Siegel RS, Brandon AR (2014). Adolescents, pregnancy, and mental health. J Pediatr Adolesc Gynecol.

[5] Barnet B, Arroyo C, Devoe M, Duggan AK (2004). Reduced school dropout rates among adolescent mothers receiving school-based prenatal care. Arch Pediatr Adolesc Med.

[6] Perper K, Peterson K, Manlove J. Diploma attainment among teen mothers. Child Trends Fact Sheet. 2010;1–4.

[7] Global SDG Indicator Platform. 3.7.2 Adolescent Birth Rate. 2021. https://sdg.tracking-progress.org/indicator/3-7-2-adolescent-birth-rate/.

[8] Wado YD, Sully EA, Mumah JN (2019). Pregnancy and early motherhood among adolescents in five east African countries: a multi-level analysis of risk and protective factors. BMC Pregnancy Childbirth.

[9] United Nations Population Fund Tanzania. Adolescent pregnancy: a review of the evidence. 2013. https://www.unfpa.org/sites/default/files/pub-pdf/ADOLESCENT%20PREGNANCY_UNFPA.pdf.

[10] United Nations Population Fund Tanzania. Fact Sheet on Teenage Pregnancy. 2018. https://tanzania.unfpa.org/en/publications/fact-sheet-teenage-pregnancy.

[11] Ajayi AI, Otukpa EO, Mwoka M, Kabiru CW, Ushie BA (2021). Adolescent sexual and reproductive health research in sub-Saharan Africa: a scoping review of substantive focus, research volume, geographic distribution and Africa-led inquiry. BMJ Glob Health.

[12] Nkata H, Teixeira R, Barros H (2019). A scoping review on sexual and reproductive health behaviors among Tanzanian adolescents. Public Health Rev.

[13] Romero-Estudillo E, González-Jiménez E, Mesa-Franco MC, García-García I. Gender-based differences in the high-risk sexual behaviours of young people aged 15–29 in Melilla (Spain): a cross-sectional study. BMC Public Health. 2014;745. 10.1186/1471-2458-14-745 PMC4118048 25053253

[14] Shimpuku Y, Madeni FE, Shimoda K, Miura S, Mwilike B (2021). Perceived differences on the role of traditional birth attendants in rural Tanzania: a qualitative study. BMC Pregnancy Childbirth.

[15] Madeni F, Horiuchi S, Iida M (2011). Evaluation of a reproductive health awareness program for adolescence in urban Tanzania—a quasi-experimental pre-test post-test research. Reprod Health.

[16] Herth K (1992). Abbreviated instrument to measure hope: development and psychometric evaluation. J Adv Nurs.

[17] Rubin DB (1987). Multiple imputation for nonresponse in surveys.

[18] Masatu MMC, Kazaura MR, Ndeki S, Mwampambe R (2009). Predictors of risky sexual behavior among adolescents in Tanzania. AIDS Behav..

[19] Kumi-Kyereme A, Awusabo-Asare K, Biddlecom A, Tanle A (2007). Influence of social connectedness, communication and monitoring on adolescent sexual activity in Ghana. Afr J Reprod Health.

[20] Babalola S, OlekoTambashe B, Vondrasek C (2005). Parental factors and sexual risk-taking among young people in Côte d’Ivoire. Afr J Reprod Health.

[21] Bastien SL, Kajula LJ, Muhwezi WW (2011). A review of studies of parent-child communication about sexuality and HIV/AIDS in sub-Saharan Africa. Reprod Health.

[22] Biddlecom A, Awusabo-Asare K, Bankole A (2009). Role of parents in adolescent sexual activity and contraceptive use in four African countries. Int Perspect Sex Reprod Health.

[23] Amoran OE, Onadeko MO, Adeniyi JD (2003). Parental influence on adolescent sexual initiation practices in Ibadan, Nigeria. Int Q Community Health Educ.

[24] Karim AM, Magnani RJ, Morgan GT, Bond KC (2003). Reproductive health risk and protective factors among unmarried youth in Ghana. Int Fam Plan Perspect.

[25] Adu-Mireku S (2003). Family communication about HIV/AIDS and sexual behaviour among senior secondary school students in Accra, Ghana. Afr Health Sci.

[26] Mmbaga EJ, Leonard F, Leyna GH (2012). Incidence and predictors of adolescent’s early sexual debut after three decades of HIV interventions in tanzania: a time to debut analysis. PLoS ONE.

[27] Wamoyi J, Fenwick A, Urassa M, Zaba B, Stones W (2010). Parent–child communication about sexual and reproductive health in rural Tanzania: implications for young people’s sexual health interventions. Reprod Health.

[28] Sakamoto K (2020). Factors influencing child survival in Tanzania: comparative analysis of diverse deprived rural villages.

[29] Schaffnit SB, Urassa M, Lawson DW (2019). “Child marriage” in context: exploring local attitudes towards early marriage in rural Tanzania. Sex Reprod Health Matters.

